# Auditory Event-Related Potentials in Antipsychotic-Free Subjects With Ultra-High-Risk State and First-Episode Psychosis

**DOI:** 10.3389/fpsyt.2019.00223

**Published:** 2019-04-15

**Authors:** Ming H. Hsieh, Yi-Ting Lin, Yi-Ling Chien, Tzung-Jeng Hwang, Hai-Gwo Hwu, Chih-Min Liu, Chen-Chung Liu

**Affiliations:** ^1^Department of Psychiatry, National Taiwan University Hospital and College of Medicine, Taipei, Taiwan; ^2^Department of Psychiatry, College of Medicine, National Taiwan University, Taipei, Taiwan; ^3^Graduate Institute of Brain and Mind Sciences, College of Medicine, National Taiwan University, Taipei, Taiwan

**Keywords:** event-related potentials, first-episode psychosis, mismatch negativity, N100, P50, schizophrenia, ultra-high risk

## Abstract

**Background:** Auditory event-related potentials (ERPs) have been utilized to study defective information processing of patients with schizophrenia. To delineate the pathophysiological processes from pre-psychotic state to first-episode psychosis, a study on subjects from ultra-high-risk (UHR) state to first-episode psychosis, ideally in an antipsychotic-free condition, can add important information to our understanding.

**Methods:** Patients with UHR state or at their first-episode psychosis (FEP) who were drug-naive or only have been temporarily treated with antipsychotics were assessed by auditory ERPs measurement, including P50/N100 (sensory gating) and duration mismatch negativity (MMN; deviance detection). A group of age-matched healthy subjects served as their controls.

**Results:** A total of 42 patients (23 UHR and 19 FEP) and 120 control subjects were recruited, including 21 pure drug-naive and 21 with very short exposure to antipsychotics. Collapsing FEP and UHR as a patient group, they exhibited significant sensory deficits manifested as larger P50 S2 amplitude, larger N100 ratio, and smaller N100 difference, and significantly less deviance detection response revealed by MMN. Such differences were less significant when treating FEP and UHR separately for comparisons. Comparisons of ERP results between drug-naive subjects and antipsychotic-short-exposure subjects revealed no significant difference in any P50/N100 and MMN parameter.

**Conclusion:** Our study is one of the few studies focused on drug-naive or minimally treated patients at pre- or early-psychotic states. Our results exhibited impaired performance in sensory gating and deviance detection shown by certain parameters. A longitudinal study with larger sample sizes will be helpful to provide more evidence to elucidate the role of antipsychotics on an individual’s neurophysiological performance at different stages of psychosis.

## Introduction

Neuroscience tools have been widely employed in schizophrenia research in recent decades (1–3). Neurobiological impairments precede the onset of a full clinical syndrome. Therefore, we can delineate psychopathological progresses by careful assessment throughout the pre-psychotic and early-psychotic states (4). Among the various neuroimaging methods, auditory event-related potentials (ERPs) have been utilized to study normal versus defective information processing of neuropsychiatric disorders, such as schizophrenia (1, 3, 5). Successful processing of sensory inputs requires two kinds of ability: sensory gating, the ability to inhibit intrinsic responses to redundant stimuli, and deviance detection, the ability to facilitate responses to less frequent salient stimuli (6). Using ERP components as measuring instruments, P50/N100 suppression represents the extent of inhibitory failure (impaired sensory gating), while MMN (mismatch negativity) indicates the magnitude of impaired deviance detection. Both processes are thought to be “pre-attentive” (passive, not demanding on subject’s active attention) and have been found to be impaired in patients with schizophrenia (3, 5, 7). Evidence suggests that auditory P50, N100, and MMN could be candidate endophenotypes of schizophrenia with intermediate relationship to susceptible genes of schizophrenia (3, 6, 8), serve as potential biomarkers to specify the progress of illness (9–12), and even help to predict if a subject would convert to full-blown psychosis (13).

As most neurobiological studies of schizophrenia were conducted in chronic patients, the possible negative impact brought by long duration of illness and long-term use of antipsychotics on brain neurochemistry and possibly on brain morphology (14) could be confounders and make it difficult to interpret those neurobiological findings (15–17). Similarly, even though P50 suppression and MMN has been regarded as endophenotypes for schizophrenia (6, 18, 19), the findings of duration MMN deficits were absent in a few studies focused on subjects at their first-episode psychosis (FEP) (7, 20–23), as well as there are studies that failed to reveal P50/N100 sensory gating deficits in this population (24, 25). However, in the studies including first-episode psychosis, whether patients were drug-free, continuing antipsychotics, or temporarily holding off antipsychotics was not all well controlled during assessment of ERPs. Ignoring such a difference in medication status may lead to confusing results (26), while administration of antipsychotics have been shown to influence ERP results, although the direction and extent of impact were diverse in different antipsychotics (27–32).

To circumvent the impact of long duration of illness and use of antipsychotics, examining subjects with drug naivety is an ideal approach. In schizophrenia research, attention has been directed towards the early state or even “pre-psychotic” state of full-blown psychosis. A lot of studies have been focused on this critical period, not only for identifying factors predicting conversion to psychosis or how to modify the trajectories of psychosis (33), but also for disentangling the complex pathogenesis of schizophrenia-related psychosis (34). The ultra-high risk (UHR), also known as late prodrome, model depicting a group of subjects who had subthreshold psychotic symptoms yet not developed full-blown psychosis (35), has been transformed into an attenuated psychosis syndrome in the *Diagnostic and Statistical Manual of Mental Disorders (DSM-5)*, Section III, as a category in need of more investigations (36). Furthermore, theoretically, Keshavan et al. pushed the model back to the beginning in the course of psychosis and named an early prodromal state with non-specific symptoms and/or basic symptoms as the “early/broadly defined at-risk mental states” (E-BARS) (37) to capture all possible features that happened during the formation of psychosis.

Our research team has started a prospective study on the psychopathological progress of the pre-psychotic state (the SOPRES study) in 2006 (38). We have recruited subjects at a gradient of clinical severities spanning from the E-BARS to UHR and FEP, together with a group of normal controls. Our ERP results of this cohort revealed a gradient of P50/N100 sensory-gating deficits across different levels of clinical severity (likely a state marker), while impaired deviance detection exhibited by duration MMNs was already detectable in people at pre-psychotic states and not much different from that in FEP (likely a trait marker) (39). But like most previous studies, the SOPRES did not control a patient’s medication status. In 2008, we initiated an open-label drug trial on UHR and first-episode psychosis, focused on those who were drug-naive or have only received a short period of antipsychotic treatment (40). The baseline assessment of this sample allows us to examine to what extent the auditory ERP components (P50/MMN/N100) will be different between subjects with UHR state and patients at first-episode psychosis, spared from the influence of antipsychotics, and compared to a large group of healthy controls.

## Methods

### Subjects

This study was approved by the Institutional Review Board of the National Taiwan University Hospital. Written informed consent was received from all participants, including written assent given by minors with informed consent from their parents. Subjects were those who participated in a 4-week open-label clinical trial using flexible dose of aripiprazole on patients with UHR state or at their first-episode psychosis between July 2008 and June 2016. Details of the clinical trial procedures have been addressed in our previous publication (40), and the definition of clinical cases is briefed below. The controls were recruited by responding to ads of various studies conducted by our schizophrenia research team with the prerequisite of having no lifetime or current psychiatric diagnosis or family history of psychotic disorders. Those who had a psychotic episode for more than 1 year, a mood episode, current use of psychoactive substance, a history of central nervous system illness or traumatic brain injury, an IQ below 70, and pregnancy were excluded from recruitment.

### Definition of Clinical Cases

The FEP subjects were those who developed full-blown psychosis that met the *Diagnostic and Statistical Manual of Mental Disorders, Fourth Edition* (*DSM-IV*) criteria for schizophrenia or schizophreniform disorder within the recent 1 year. The UHR subjects presented subthreshold psychotic symptoms meeting the comprehensive assessment of at-risk mental status criteria (41) either with attenuated psychotic symptoms or with brief limited intermittent psychotic symptoms. Subjects have never received antipsychotic treatment before were designated as the “drug-naive” group. Subjects reported to have received a known antipsychotic or psychotropic agent that exerted an effect or adverse event very likely to be associated with antipsychotics for a total of less than 3 months were designated as the “antipsychotic-short-exposure” group.

The antipsychotic-short-exposure group was asked to remain antipsychotic-free for at least 1 week before baseline assessments. Patient’s clinical severity was assessed by a Mandarin version of the Positive and Negative Syndrome Scale (PANSS) for schizophrenia and received ERP studies at baseline and 4 weeks after completing treatment with aripiprazole. In this paper, we focused on their baseline ERP results that were not affected by antipsychotic treatment.

### Testing Environment

Before ERP recording, audiometry testing was used to exclude subjects who could not detect 40-dB sound pressure level tones at 500, 1,000, and 6,000 Hz presented to either ear. The standard procedures for auditory P50/N100 and MMN paradigm were based on established protocols (42–45). The participants had not smoked for at least 1 h before sessions (46) and were asked to lie down in supine position in a comfortable recliner in a sound-attenuating, electrically shielded booth and instructed to relax with his/her eyes open and to focus on a fixation point (P50 and N100 session) or a cartoon running with no sound on the video monitor (MMN session). There were no tasks performed during the test. During the testing, electroencephalography and stimuli would be recorded continuously, and subjects were closely observed through a video monitor. They would be monitored visually and by electroencephalography (EEG) for signs of sleep or slow wave activity, which, if present, prompted the experimenter to speak briefly with the subject.

The EEG signals were recorded with a Quik-Cap (Compumedics Neuroscan, El Paso, TX, USA) from 32 scalp locations. According to the Quik-Cap website, all electrodes were placed according to the International 10–20 electrode placement standard. Electrodes placed at the tip of the nose and at Fpz served as the reference and ground, respectively. Four additional electrodes were located above and below the left eye and at the outer canthi of both eyes to monitor blinks and eye movements. All electrode impedances were kept below 5 kΩ prior to recording.

### Stimuli Session and ERP Recording

The auditory stimuli were generated by a Neuroscan STIM system, and data were recorded on a Neuroscan ACQUIRE system (Compumedics Neuroscan, El Paso, TX, USA). Stimuli were digitized at a rate of 1 kHz, and an online band-pass filter at 0.5–100 Hz, without 60-Hz notch filter, was applied. Auditory stimuli were presented to the subjects binaurally* via *foam insert earphones in two consecutive sessions, i.e., the session of paired-click paradigm for P50/N100 followed by the duration MMN session.

Online averaging was used to monitor the number of trials free from gross artifacts (defined as activities exceeding ±100 μV in the −100 to 500 ms time window following stimuli). Regarding the paired-click P50/N100 paradigm, paired auditory clicks (1 ms, 85 dB) were presented every 8–12 s through the whole test session (average: 10 s), with a 500-ms interstimulus interval (47, 48). The paired-click P50/N100 session was terminated when a minimum of 120 artifact-free trials had been obtained, which took about 30 min. For the duration MMN paradigm, pure tone stimuli (1 kHz, 85-dB SPL, 5-ms rise/fall) were generated by the Neuroscan STIM system. The auditory stimuli consisted of standard stimuli (90%, 50-ms duration) and deviant stimuli (10%, 100-ms duration) delivered in a pseudo-random order with the constraint that deviant stimuli could not be repeated back to back. The cartoon soundtrack was turned off and replaced by the experimental auditory stimuli that were presented at a fixed 500-ms onset-to-onset asynchrony. The MMN session was continued until a minimum of 225 artifact-free deviant trials had been collected online, which took approximately 30 min.

### Offline Data Processing

Details regarding offline signal analysis, using Neuroscan Edit 4.5 software (Compumedics Neuroscan, El Paso, TX, USA), were followed as our previous publications (39, 44, 49). All data were processed by researchers who were blind to the subject’s group (50). Semi-automated procedures using the Tool Command batch processing language (TCL) began with electrooculography (EOG) artifact reduction through a built-in pattern-recognition algorithm (51). For P50/N100, the data were epoched for the time window from −100 to 923 ms of the first click, covering both S1 and S2 in the same epoch. All epochs containing activities exceeding ±50 μV were excluded. To prevent temporal aliasing, epochs were averaged and digitally band-pass-filtered (10 to 50 Hz for P50, 1 to 50 Hz for N100) in the frequency domain. Trials with artifacts were manually rejected. By using preset intervals, peaks and preceding troughs were then automatically detected at the Cz electrode. The P50 peak was defined as the largest positive deﬂection between 45- and 75-ms poststimulus, and its amplitude was assessed as the difference between this peak and the preceding negative trough (not earlier than 30-ms poststimulus). The N100 component was identified as the most negative deﬂection within 80- to 150-ms poststimulus, and N100 amplitude was defined as the absolute difference between the N100 peak and the preceding positive trough. P50 and N100 parameters included the S1 amplitude, S2 amplitude, amplitude difference (S1 − S2), and P50/N100 gating ratio (S2/S1). A maximum gating ratio of 2 was used to prevent outliers from disproportionately affecting the group means (39, 44, 52).

For duration MMN analysis, each subject’s continuous data file to 500-ms poststimulus. EEG responses to standard and deviant stimuli were separately averaged to create a standard ERP and a deviant ERP, and both were low-pass filtered at 20 Hz (0-phase shift and 24-dB/octave roll-off) to remove any residual high-frequency artifacts. MMN waveforms were generated by subtracting the standard ERP from the deviant ERP. MMN indices were measured as the mean voltage from 135 to 205 ms of the Fz electrode (18, 39, 53–55).

### Statistical Analysis

Statistical analyses were performed using SPSS v16.0 software (SPSS, Chicago, IL). For demographic characteristics and ERP parameters, the results are presented in means and standard deviations (±SD). Chi-square tests were used for categorical variables. Putting subjects with UHR state and first-episode psychosis together as a patient group, we compare control vs. patient group in demographics and ERP results. In addition, comparison between controls, UHR, and FEP groups with analysis of variance (ANOVA) was performed, and we also calculated comparison between control/drug-naive/antipsychotic-short-exposure groups. All *post hoc* comparisons were made using the Scheffe test. Statistical significance was set at *p* < 0.05. Cohen’s *d* effect size was calculated for all ERP parameters.

## Results

A total of 42 patients (19 FEP and 23 UHR) and 120 control subjects were recruited. Among them, 21 patients endorsed pure drug naivety (7 FEP and 14 UHR), and the other 21 patients (12 FEP and 9 UHR) have only been exposed to antipsychotics for no longer than 3 months. Indeed, the majority of these 21 short-exposure patients took antipsychotics at a low dose level no longer than 4 weeks and they could endure a washout period of 1 week prior to receiving ERP assessment with no apparent worsening of symptoms. Both paired-click P50/N100 paradigm and duration MMN paradigm took about 30 min in duration. Although all 42 patients had ERP recorded, 9 patients (3 UHR/6 FEP) could not finish the P50/N100 paradigm, while 8 patients (5 UHR/3 FEP) could not tolerate duration MMN paradigm, yielding the numbers of subjects with data available for further analysis to be 33 and 34 for P50/N100 and MMN, respectively.

### Demographic and Clinical Characteristics

In **Table 1**, UHR and FEP were treated collectively as a patient group to compare with the control group, while in **Table 2**, UHR and FEP were examined separately for any difference between these two groups. There were no statistical differences in age and gender when the patient group is compared to the control group, although the UHR group was significantly younger than the FEP group (23.64 ± 5.08 vs. 28.45 ± 8.33, *p* = 0.022). The controls had 1.6 years more in education and reported much lower amount of smoking compared to the patient group, while there was no difference in these two variables between the UHR and FEP groups. In terms of clinical severity shown by PANSS scores, the UHR patients only exhibited lower scores in positive symptom subscales than the FEP patients (15.0 ± 2.9 vs. 19.4 ± 4.6, *p* < 0.001), while their scores in negative symptoms and general symptoms subscales were comparable to each other.

**Table 1:** Demographics and ERP results of control and patient groups (SD in parentheses).^a^

	Control	Patients	Statistics	Effect size (Cohen’s *d*)
	*n* = 120	*n* = 42		
Age	26.63 (5.09)	25.82 (7.08)	0.424	
Male gender (%)^b^	63 (52.5%)	21 (50%)	χ^2^ = 0.08, *p* = 0.78	
Education (years)	15.62 (1.88)	14.00 (2.51)	<0.001**	
Smoking PPD	0.03 (0.1)	0.15 (0.4)	0.035*	
PANSS				
Positive symptoms (P1 to P7)	—	17.0 (4.3)		
Negative symptoms (N1 to N7)	—	14.1 (5.9)		
General psychopathology (G1 to G16)	—	35.3 (8.6)		
				
MMN Fz	−1.36 (0.81)	−1.05 (0.78)	0.047*	0.39
P50				
S1 amplitude	2.44 (1.06)	2.53 (1.4)	0.679	0.07
S2 amplitude	1.09 (0.64)	1.45 (0.84)	0.008**	0.48
P50 ratio	0.51 (0.34)	0.63 (0.38)	0.075	0.33
P50 difference	1.35 (1.07)	1.08 (1.39)	0.239	0.22
N100				
S1 amplitude	6.73 (3.27)	5.82 (2.95)	0.150	0.29
S2 amplitude	2 (1.31)	2.46 (1.3)	0.073	0.35
N100 ratio	0.36 (0.31)	0.51 (0.34)	0.017*	0.46
N100 difference	4.73 (3.35)	3.36 (2.62)	0.030*	0.46

UHR, ultra-high-risk group; FEP, first-episode psychosis group. ERP, event-related potential. MMN, mismatch negativity.

a Some subjects failed to stay before the ERP session was terminated. The number of analyzable P50/N100 subjects was 20 UHR and 13 FEP. The number of analyzable MMN subjects was 18 UHR and 16 FEP.

b Chi-square tests.

* p < 0.05.

** p < 0.01.

**Table 2:** Demographics and ERP results of three groups (SD in parentheses).^a^

	Control	UHR	FEP	Statistics	
				*Post hoc* Scheffe *p* values	Effect size (Cohen’s *d*)
	A (*n* = 120)	B (*n* = 23)	C (*n* = 19)		
Age	26.63 (5.09)	23.64 (5.08)	28.45 (8.33)	A vs. B: 0.064 A vs. C: 0.417 B vs. C: 0.022*	
Male gender (%)^b^	63 (52.5%)	14 (60.9%)	7 (36.8%)	χ^2^ = 2.48, *p* = 0.29	
Education (years)	15.62 (1.88)	13.74 (2.83)	14.32 (2.08)	A vs. B: 0.000** A vs. C: 0.040* B vs. C: 0.6655	
Smoking PPD	0.03 (0.1)	0.15 (0.4)	0.16 (0.4)	A vs. B: 0.044* A vs. C: 0.057 B vs. C: 0.997	
PANSS					
Positive symptoms (P1 to P7)		15.0 (2.9)	19.4 (4.6)	B vs. C: 0.001**	1.14
Negative symptoms (N1 to N7)		14.2 (5.5)	14.0 (6.5)	B vs. C: 0.923	0.03
General symptoms (G1 to G16)		35.4 (8.4)	35.6 (9.1)	B vs. C: 0.923	0.02
MMN Fz	−1.36 (0.81)	−0.99 (0.88)	−1.11 (0.68)	A vs. B: 0.195 A vs. C: 0.517 B vs. C: 0.905	0.44 0.33 0.15
P50					
S1 amplitude	2.44 (1.06)	2.83 (1.59)	2.07 (0.89)	A vs. B: 0.366 A vs. C: 0.550 B vs. C: 0.180	0.07 0.38 0.59
S2 amplitude	1.09 (0.64)	1.48 (0.8)	1.41 (0.93)	A vs. B: 0.071 A vs. C: 0.280 B vs. C: 0.967	0.54 0.4 0.08
P50 ratio	0.51 (0.34)	0.58 (0.34)	0.71 (0.42)	A vs. B: 0.706 A vs. C: 0.139 B vs. C: 0.563	0.21 0.52 0.34
P50 difference	1.35 (1.07)	1.35 (1.53)	.66 (1.05)	A vs. B: 1 A vs. C: 0.125 B vs. C: 0.242	0 0.65 0.52
N100					
S1 amplitude	6.73 (3.27)	6.84 (2.92)	4.25 (2.3)	A vs. B: 0.991 A vs. C: 0.030* B vs. C: 0.076	0.04 0.88 0.99
S2 amplitude	2 (1.31)	2.81 (1.1)	1.93 (1.43)	A vs. B: 0.037* A vs. C: 0.982 B vs. C: 0.164	0.67 0.05 0.69
N100 ratio	0.36 (0.31)	0.48 (0.29)	0.55 (0.41)	A vs. B: 0.282 A vs. C: 0.118 B vs. C: 0.824	0.40 0.52 0.20
N100 difference	4.73 (3.35)	4.02 (2.48)	2.33 (2.58)	A vs. B: 0.657 A vs. C: 0.038* B vs. C: 0.331	0.24 0.80 0.67

UHR, ultra-high-risk group; FEP, first-episode psychosis group.

a Some subjects failed to stay before the ERP session was terminated. The number of analyzable P50/N100 subjects was 20 UHR and 13 FEP. The number of analyzable MMN subjects was 18 UHR and 16 FEP.

b Chi-square tests.

* p < 0.05.

** p < 0.01.

### Comparisons of Event-Related Potentials

**Figure 1:**
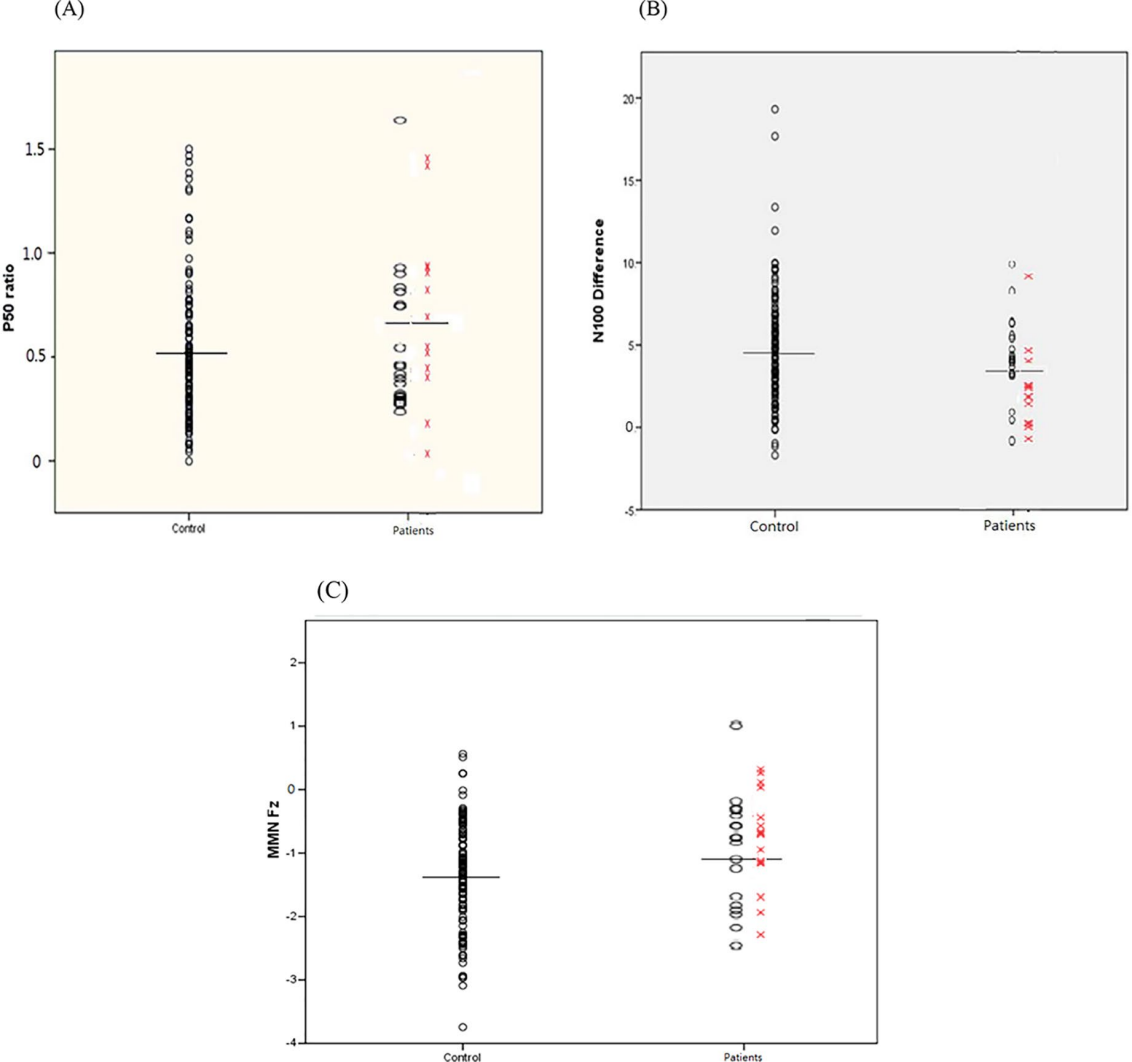
P50 ratios (S2 amplitude/S1 amplitude) **(A)**, N100 differences (μV; S2 amplitude − S1 amplitude) **(B)**, and mismatch negativity (MMN) at electrode Fz **(C)** of individual participants between groups. The horizontal lines indicate the mean values within control vs. patient group, while the patient group consists of ultra-high-risk (UHR; oval) and first-episode psychosis (FEP; X) subjects. For P50 and N100, a larger ratio (S2/S1) and a smaller difference (S1 − S2) indicate poorer sensory gating. For MMN, a larger (less negative) value indicates poor deviance detection.

**Figure 2:**
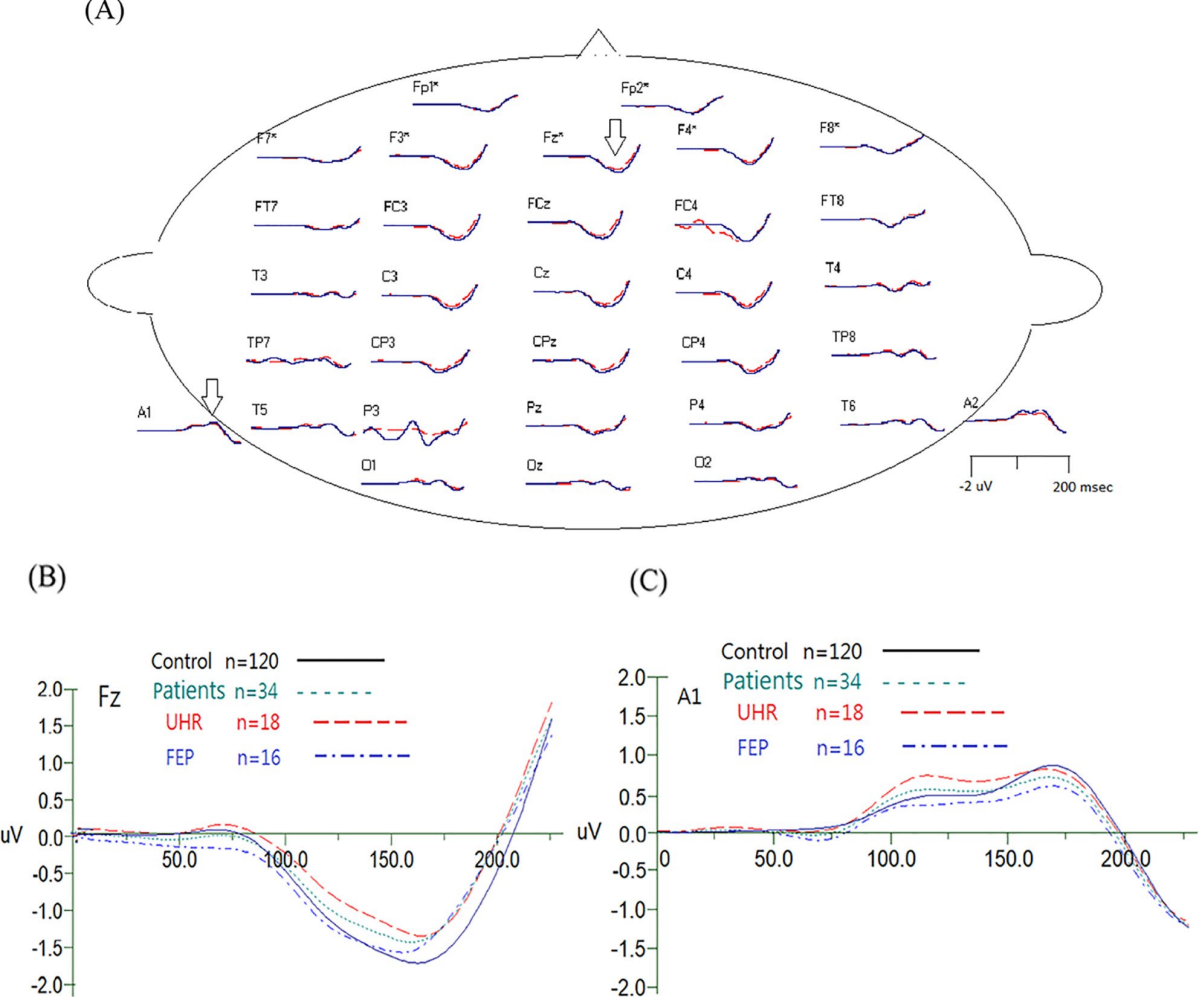
**(A)** Demonstrated grand average MMN waveforms for healthy control subjects (in blue) and patients (in red). The arrows indicated the waveform reversed in polarity at the mastoid electrodes, which is typical for MMN. A close-up of grand average waveform at electrode Fz and A1 (mastoid) electrodes is shown in **(B)** and **(C)**.

Also presented in **Table 1**, the patient group had a smaller magnitude in MMN, a larger P50 S2 amplitude, a larger N100 amplitude ratio, and a smaller N100 difference compared to the control group. However, as detailed in **Table 2**, the patient group’s smaller amplitude of MMN was not so evident when pairwise comparisons were made between UHR and controls as well as between FEP and controls. Similarly, when UHR and FEP were compared to the control group separately, no significant difference could be found in P50 parameters. The only significant differences remained in N100-related parameters: the FEP had a significant lower amplitude in N100 S1 amplitude compared to the controls, and the UHR had a higher N100 S2 amplitude than the controls, while the larger N100 amplitude ratio became insignificant in both groups, but the N100 difference remained significantly smaller in the FEP group but not in the UHR group. Comparisons between control subjects and patients in P50 ratios, N100 differences and MMN values are shown in **Figure 1**. The average MMN waveforms are demonstrated in **Figure 2**.

A head-to-head comparison of ERP results between the drug-naive subjects and the antipsychotic-short-exposure subjects is shown in **Table 3**. Apparently, there was no significant difference in any P50/N100 and MMN parameter between these two groups.

**Table 3:** ERP results of control/drug-naive/antipsychotic-short-exposure groups (SD in parentheses).^a^

	Control (*n* = 120)	Drug-naive (*n* = 21)	Antipsychotic short exposure (*n* = 21)	Statistics	
				*Post hoc* Scheffe *p* values	Effect size (Cohen’s *d*)
	A	B	C		
MMN Fz	−1.36 (0.81)	−1.08 (0.88)	−1.00 (0.67)	A vs. B: 0.396 A vs. C: 0.267 B vs. C: 0.967	0.34 0.45 0.10
P50					
S1 amplitude	2.44 (1.06)	2.77 (1.77)	2.33 (1.00)	A vs. B: 0.572 A vs. C: 0.936 B vs. C: 0.551	0.29 0.10 0.31
S2 amplitude^b^	1.09 (0.64)	1.32 (0.56)	1.56 (1.02)	A vs. B: 0.480 A vs. C: 0.027^b^ B vs. C: 0.600	0.36 0.67 0.28
P50 ratio	0.51 (0.34)	0.54 (0.24)	0.71 (0.45)	A vs. B: 0.942 A vs. C: 0.083 B vs. C: 0.402	0.09 0.56 0.46
P50 difference	1.35 (1.07)	1.45 (1.51)	0.77 (1.23)	A vs. B: 0.947 A vs. C: 0.141 B vs. C: 0.239	0.09 0.53 0.50
N100					
S1 amplitude	6.73 (3.27)	6.06 (3.01)	5.61 (2.96)	A vs. B: 0.751 A vs. C: 0.391 B vs. C: 0.923	0.21 0.35 0.15
S2 amplitude	2 (1.31)	2.64 (0.92)	2.32 (1.56)	A vs. B: 0.209 A vs. C: 0.631 B vs. C: 0.783	0.50 0.24 0.24
N100 ratio	0.36 (0.31)	0.50 (0.21)	0.52 (0.42)	A vs. B: 0.270 A vs. C: 0.149 B vs. C: 0.989	0.47 0.49 0.06
N100 difference	4.73 (3.35)	3.43 (2.40)	3.30 (2.86)	A vs. B: 0.336 A vs. C: 0.214 B vs. C: 0.993	0.40 0.43 0.05

a Some subjects failed to stay before the ERP session was terminated. The number of analyzable P50/N100 subjects was 15 for drug-naive and 18 for antipsychotic short exposure. The number of analyzable MMN subjects was 18 for drug-naive and 16 for antipsychotic short exposure.

b Post hoc Scheffe test revealed significant differences between control and antipsychotic-short-exposure groups (p = 0.027).

## Discussion

It is believed that clinical and cognitive deficits of psychosis may be due to dysfunction at the earlier stages of information processing (56). Bora and Murray’s meta-analysis highlighted that cognitive deficits are already established before the prodromal phases of psychosis (57), compatible with our previous publication regarding neurocognitive performance in different stages of pre- and early-psychotic states (58). Such neurocognitive disturbance might represent different components of auditory modality in sensory processing dysfunctions in schizophrenia, and our neurophysiological paradigms measuring “pre-attentive, passive” auditory ERPs in UHR and first-episode psychosis subjects can add valuable information to this field (59).

Although many studies of MMN were conducted on subjects with UHR states, only few have also measured P50/N100 in the same study (60). Also, several studies have included patients with first-episode psychosis and examined them separately from chronic schizophrenia, and most publications reported auditory pre-attentive (passive) ERPs after the patients had been treated with antipsychotics. For example, Koshiyama et al. investigated duration vs. frequency MMN in 14 FEP patients, 16 UHR individuals, and 16 healthy controls. They concluded that duration MMN is superior to frequency MMN as a trait marker in the early stages of psychosis, and a smaller duration MMN amplitude in early stages of psychosis may reflect altered developmental process rather than progressive brain pathology (61). However, most of their patients with either FEP or UHR have been treated with antipsychotic medication prior to the experiment, leaving a possible confounder in their interpretation of results.

As Haigh et al.’s meta-analysis of MMN in first-episode schizophrenia patients highlighted a need to conduct study on medication-naive individuals (26), our report is one of the few studies focused on P50/N100/duration MMN in drug-naive or minimally treated FEP and UHR patients. Consistent with our previous report when drug naivety was not strictly defined in that study population, a linear trend of more deviance from controls across different levels of clinical severity was noticed in P50 ratios (S2/S1) and N100 differences, even though the differences in P50 and N100 between control and clinical groups were not statistically significant (39). Specific to study on sensory gating adopting P50/N100 paradigms, our findings are in line with Shaikh et al.’s 36 unmedicated patients who met attenuated psychosis syndrome (equivalent to our UHR) and have already exhibited P50 sensory gating deficits at this pre-psychotic state (62). Similarly, Brockhaus-Dumke et al. found impaired P50 suppression (S2/S1 ratio) in all clinical severities (at risk, true prodromal, first episode, and chronic schizophrenia), while impaired N100 suppression (S1 − S2 difference) was also seen in all clinical groups except in the at-risk subjects (63); the latter is exactly the same with our finding. Specific to studies on MMN, our results are similar to Mondragon-Maya et al.’s (23) and During et al.’s (64) MMN and P3a studies, which revealed no impaired deviance detection ability among antipsychotic naive first-episode psychosis patients and individuals at clinical high risk for psychosis and control subjects.

In addition to verifying previous studies, we took a closer look into our findings. When UHR and FEP were compared to normal controls separately, the directions of changes of ERP parameters are of great interest. Based on the sensory gating failure theory, the patients are expected to reveal smaller S1 and larger S2 in P50 signals. This pattern could only be seen in our FEP subjects but not the UHR patients, while the latter exhibited larger, but not smaller, S1, together with larger S2. Although none of these findings reached statistical significance, our findings derived from subjects not confounded by antipsychotic medication might give a hint to understand the dynamic changes of sensory gating in patients with schizophrenia during the progress of their illness. Also, even though no difference in MMN could be detected when UHR and FEP were compared to normal controls separately, collectively as a patient group, their MMN deviance detection ability is lower than that of normal controls, also a finding not confounded by antipsychotics.

Two major limitations of the current study are worth noting. The relatively small sample size of the UHR (*n* = 23) and FEP (*n* = 19) groups limits our statistical power to detect smaller differences between groups, such as dividing the pure drug-naive UHR and FEP from those who had short exposure to antipsychotics in either group, but we believe that the majority of our participants had limited impact by antipsychotic treatment, comprising a very valuable sample. Future studies recruiting a larger sample would be necessary to verify our findings. Second, none of our UHR patients converted to full-blown psychosis during a period of 4 weeks. We did not know how many of them would eventually develop psychosis after 1 or 2 years, while previous studies suggested that ERP performance of the converters were likely worse than that of the nonconverters (11, 13, 65).

In summary, our ERP results of antipsychotic-free subjects with UHR state and first-episode psychosis are not much different from those studies that did not control antipsychotic medication status. Our drug-naive subjects showed no significant difference from their antipsychotic-short-exposure counterparts as well. If this is true, it will be convenient to use this modality to measure patient’s sensory gating performance regardless of the impact of antipsychotics, at least at the pre- and early-psychotic states. Nonetheless, we will examine if there are differences in ERP performance between baseline and by the end of a 4-week exposure to antipsychotic treatment. A longer follow-up of prospective longitudinal study will be helpful to provide more evidence to elucidate the role of antipsychotic medication on an individual’s neurophysiological performance at different stages of psychosis.

## Ethics Statement

This study was carried out in accordance with the recommendations of the guidelines of National Taiwan University Hospital Research Ethics Committee with written informed consent from all subjects. All subjects gave written informed consent, including written assent given by minors with informed consent from their parents in accordance with the Declaration of Helsinki. The protocol was approved by the Research Ethics Committee of National Taiwan University Hospital.

## Author Contributions

MHH and C-ML reviewed literature and designed this study. MHH and Y-TL did the ERP study and data analysis. Y-LC performed the statistics. C-ML oversaw the clinical trial. C-CL, T-JH and H-GH handled the early psychosis studies. All authors have helped to recruit subjects and involved in clinical and diagnostic assessments. MHH wrote the first draft of the manuscript. C-CL finalized the writing and editing of the manuscript. All authors contributed to and have approved the final manuscript.

## Funding

This work was supported by the Ministry of Science and Technology (Grant Number MOST 103-2314-B-002-020-MY2, 106-2314-B-002-236-, and 107-2314-B-002-012-) and the National Science Council, Taiwan (Grant Numbers NSC 95-2221-E-002-028 and NSC 98-2314-B- 002-047-MY3).

## Conflict of Interest Statement

The authors declare that the research was conducted in the absence of any commercial or financial relationships that could be construed as a potential conflict of interest.
